# Physician induced demand for knee replacement surgery in Iran

**DOI:** 10.1186/s12913-021-06697-6

**Published:** 2021-08-02

**Authors:** Cyrus Alinia, Amirhossein Takian, Nasser Saravi, Hasan Yusefzadeh, Bakhtiar Piroozi, Alireza Olyaeemanesh

**Affiliations:** 1 Department of Health Economics and Management, School of Public Health, Urmia University of Medical Sciences, Urmia, Iran; 2 Health Equity Research Center (HERC), Tehran University of Medical Sciences, Tehran, Iran; 3 Department of Global Health and Public Policy, School of Public Health, Tehran University of Medical Sciences, Tehran, Iran; 4 Department of Health Management and Economics, School of Public Health, Tehran University of Medical Sciences, Tehran, Iran; 5 Health Insurance Research Center, Armed Forces Medical Service Insurance Organization (AFMSIO), Tehran, Iran; 6 Social Determinants of Health Research Center, Research Institute for Health Development, Kurdistan University of Medical Sciences, Sanandaj, Iran; 7 Department of Health Economics, National Institute for Health Research (NIHR), Tehran University of Medical Sciences, Tehran, Iran

**Keywords:** Knee replacement surgery, Physician induced demand, Iran, Panel data, GMM

## Abstract

**Background:**

The structure of the Iranian health system has raised this hypothesis that a part of the Knee Replacement Surgery (KRS) services are provided due to Physician-Induced Demand (PID).

**Methods:**

This paper used an unbalanced individual panel data covering the steady-state 15,729 KRSs performed by 995 surgeons provided by the Armed Forces Insurance Organization at the provincial level over the 60 months (2014–2018). We use a generalized method of moment’s system (GMM-SYS) to obtain consistent and asymptotically efficient estimates, which provide a vital instrument for our dynamic panel data.

**Results:**

The outcomes show that with unequal increasing orthopedic surgeons to population ratio, both the number and size of KRS services were increased significantly at a 1 % level. Given that the positive elasticity obtained for the service size was significantly larger than the number of services, the findings give strong support for the existence of PID in the Iran system for KRS care. Also, the raw and population-adjusted number of KRS, cost, and the surgery per active physician increased significantly at the monthly province level.

**Conclusions:**

This is the first time that the existence of PID in the Iranian health system is investigated using approved econometric models. The findings indicate that the health system structure has been provided the conditions for aggressive, costly, and high-risk services such as KRS to be exposed to PID.

## Background

Knee osteoarthritis is a chronic and age-related condition associated with pain and disability, and around 10 % of men and 13 % of women over the age of 60 suffer its typical symptoms [1]. The disease imposes significant physical limitations on the patients and causes a loss of 19 and 34 % of health-related quality of life, respectively, in moderate and severe cases, on average [2]. The prevalence of knee osteoarthritis disease in rural and urban areas of Iran is 19.3 and 15.3 %, respectively [3, 4], which is more prevalent among women [5].

Starting in the 1970 s, Knee Replacement Surgery (KRS) is an effective and expensive approach for end-stage knee arthritis [6]. The demand for this treatment method is increasing substantially. The rate of person-year in the United States has more than seven times over the past four decades [7]. Its population-adjusted rate in Iran has doubled in the last five years. The mean age of patients undergoing KRS in Iran is reported about 65 years [8–10], lower than in developed countries [6, 11].

Increasing population age and obesity are introduced as the most important reasons for performing KRS [12]. However, given that both the factors do not change instantly at the population level but show significant changes gradually over time, the increased demand for this service over shorter periods could have other structural reasons; technology advances, increasing the level of community health knowledge, increasing the level of community income, changing lifestyles, and increasing access to services due to the entry of more orthopedic surgeons into the health market [13–15]. Due to the considerable information asymmetry between the surgeon and the patient, the surgeons simultaneously act as suppliers (as service providers) and demanders (as patient agents). This means that surgeons are entirely free to prescribe the type of treatment, providing conditions for an imperfect agency problem. In such cases, the decision to prescribe the service is influenced by the economic motivations of the providers, and the surgeon prioritizes her/his preferences over the patient [16].

As such, the question raised by health policymakers is whether the KRS prescribed by the surgeons were based on the patient’s needs or not? This question becomes especially acute when the physician to population ratio has increased over time and the physician’s share of patient decreases and naturally reduces his/her income. Physician Induced Demand (PID) is seen to compensate for lost income due to fewer patients. However, this solution is more suitable for diagnostic and elective surgery services [17].

The existence of PID in the Iranian health system is very likely for structural reasons. Physicians have a very high degree of freedom of action in the Iranian health system, and regulatory bodies do not effectively monitor their performance. Reimbursement to the providers is in the form of Fee-For-Service and dramatically increases the motivation of providers to deliver more services. Also, the physician-population ratio has increased significantly over the past decade [18].

Various types of health insurance cover more than 90 % of Iran’s population. Social Security Organization, Health Insurance Organization, and Armed Forces Insurance, with the coverage rates of 42.87 %, 42.80 %, and 5.00 %, respectively, are the largest Iran’s health insurance organizations. The first two insurances are committed to providing services to Iranian workers, the underprivileged, government employees, and rural residents, covering 90 % of inpatient services and 70 % of outpatient services in public health facilities. Also, the Armed Forces Insurance is committed to providing outpatient and inpatient services to the Armed Forces and their families, most of whose services are free of charge in government and military centers and offset between 65 and 90 % of private-sector costs [18–20].

Using unbalanced panel data covering KRS services provided by the Armed Forces Insurance Organization at the provincial level for 60 months, this study has designed econometric modeling to answer the above question and fill this knowledge gap.

## Methods

### Dataset

We use microeconomic data for the monthly average number of KRS activities by each orthopedic specialist over 2014 to 2018, compiled from the Iranian Armed Forces Insurance Organization at the provincial level. The unbalanced individual panel data covers the steady-state 15,729 surgeries performed by 995 surgeons. These suppliers satisfy this condition that began their activity before 2014 and had not been retired during the study time. Population data, including population size of people older than 50 years and average monthly income for each (province) and each (month) are extracted from the census results for 2011 and 2016 years. To estimate the population size of the middle years, the constant annual multiplier of 0.021 was used as the average annual growth.

The economic theory of supply and demand explains the presence of PID for KRS when an increased number of the surgeons decreases the patient’s quota for each The economic theory of supply and demand explains the presence of PID for KRS when an increased number of surgeons decreases the patient’s quota for each surgeon, thereby lead to reducing the number of surgeries and his/her income. Therefore, within the context of fixed prices, the increase in supply has led to imbalance, and in order to achieve equilibrium, doctors are trying to regain their level of income in two ways; first, increase the number of surgeries, and second, increase the price of each surgery by changing the type of surgery and making it more expensive. Ultimately, an increase in supply leads to an increase in demand [17, 21]. Part of this elevated demand has been increased access to the services by increasing the household’s income, improving the level of health insurance coverage, the emergence of new advanced technologies, raising the level of public health awareness, or increasing the elderly population. However, according to the theory, part of this increase in demand could be due to PID. So, the induced KSR should be measured as the increment in the activities that would not have performed whiteout training and employing more orthopedic surgeons. Therefore, in the current study, the changes in the supply of KRS services are measured by the density variable (${d}_{it}$) of orthopedic surgeons, which is equal to the ratio of surgeons to every 100,000 population over 50 years of age.

### Variables

Following Delattre and Dormont [22], to estimating the potential PID for KRS, we require measuring the activities of the surgeons in the two following ways:

- $n_{it}$:the number of performed KRS by each physician at province $i$ at month $t$. This variable can show changes in the number of surgeries over time, but has four main drawbacks; First, it is not able to identify the access effect for cases that had an unmet need for any reason, including the inability to purchase the services, lack of geographical access to the surgeon, lack of knowledge about the existence of treatment and the like. Second, it does not show the content of the service. As mentioned earlier, the physician can manipulate the overall cost of the service by changing the type and quality of service, but ${n}_{it}$ variable cannot measure it. Third, it does not depict the initiating effect. If the patient asked the surgery, this request should not be considered a PID, even if the operation was unnecessary. Fourth, it does not consider the practice style of physicians. Different surgeons do not necessarily make the same decision for a particular patient regarding whether or not to perform the procedure and service type, so the procedure alone should not be measured as induced demand. However, the ${n}_{it}$ variable is a crucial factor in measuring PID in this study.
- $s_{it}$: the size of performed KRS by each physician at province $i$ at month $t$. This variable is obtained by multiplying the number of operations performed by the cost of each operation, which is paid by the patient and the health insurance; ${s}_{it}={n}_{it}{f}_{it}$. A significant increase in the mean of this variable over time clearly indicates a change in the type of operation, a rise in the price of the material used, or a combination of both. Given the ${s}_{it}$ can simultaneously measure the surgeries’ amount and content, it can eliminate all four weaknesses of the previous variable. Even if the service would be patient-initiated, the decision about the intensity of the service is made by the surgeon. So, increasing the mean cost of each KRS over time cannot be due to increased levels of access to the service, and also the relative value of the cost of each surgery eliminates the constant effects of the providers’ behavior, such as physicians’ preferences and practice style.

The variables ${n}_{it}$ and ${s}_{it}$ represent the demand for the service, and their changes indicate the demand shock. In a supply-demand framework, the study of changes in each of these variables in response to changes in service supply defined by the ratio of surgeons to the elderly population, ${d}_{it}$, with controlling other influential factors, can show the existence of induced demand. Increasing the ${d}_{it}$causes a positive shift in the total supply curve and exits from the initial equilibrium point. Due to the fixed prices, the new supply and demand curves intersect at the disequilibrium price. According to the logic of Delattre and Dormont [22] and Sorensen and grytten [23], if physicians do not induce the KRS, supply rationing will arise, the patient quota of each physician will decrease, and therefore the microeconomic elasticity of ${n}_{it}$ will be negative. On the other hand, if surgeons increase the size of the service (${s}_{it}$), in response to the supply rationing, they can partially compensate for their lost income. Given that no managed care exists in Iran, surgeons can freely increase the ${s}_{it}$. Therefore, the sufficient conditions for the existence of PID for KRS are; the elasticity of ${s}_{it}$will be positive and greater than the elasticity of ${n}_{it}$.

### The models

This section presents a dynamic panel data (DPD) model for investigating the induced demand in KRS in Iran. The specification is in accordance with the model performed by Delattre and Dormont [22], which relates to the investigation on the existence of PID for French physicians. This model makes it possible to an induced demand estimation applying the elasticity of the KRS in response to changing physician density. Besides, DPD allows us to consider both random and permanent unobserved heterogeneities related to the characteristics of the supplier and demanders.

This is especially important when unobserved factors are correlated with independent variables, which can induce bias in estimation. The most important constant and permanent feature of physicians in determining service delivery is their practice style that our model can consider. The presence of lagged dependent variables in the model puts our regression inside the context of dynamic panel models. All variables are transformed to logarithm forms so that coefficients may be interpreted as elasticity.

To test the PID, we need three separate econometric models that are exactly the same, but their dependent variables include ${n}_{it}$, ${S}_{it}$, and ${q}_{it}$:

1 $$\log\left(n_{it}\right)=\alpha\log\left(d_{it}\right)+\gamma\log\left(inc_{it}\right)+\mathrm Z'_{t\left[1,k\right]}\;\theta_{\left[k,1\right]}+\xi_\delta+v_i+\varepsilon_{it}$$

$$t=m4-2014,\dots , m3-2019;i=\text{1,2}, \dots , 22$$

The constants ${ {\upnu }}_{i}$ denotes for those fixed and inherent characteristics of the patients in each province that are not obviously considered in the model, including gender, age, disease severity, level of insurance coverage, level of earning, and selecting the physicians based on their reputation. Given that ${d}_{it}$is an aggregate variable because it shows the intensity of competition between orthopedic surgeons to perform KRS at the provincial level, presence of a random term ${{\upxi }}_{{\updelta }}$ in the perturbation is recommended. The number and type of KRSs also depends on time-varying determinants such as the development and supply of new technologies, economic growth, changes in demographic characteristics, and lifestyle changes that affect all surgeons alike. The sum of these factors is denoted by ${\text{Z}}_{\text{t}\left[1,\text{k}\right]}^{{\prime }}$. Also, in the one-step estimator, the error term ${{\upepsilon }}_{it}$ is assumed to be i.i.d. (0, ${{\updelta }}_{\epsilon }^{2}$) across provinces and time. In the two-step estimator, the residuals of the first step are applied to consistently estimate the variance-covariance matrix of the perturbation ${{\upepsilon }}_{it}$, relaxing the homoscedasticity assumption.

The constant effects of ${ {\upnu }}_{i}$and ${{\upxi }}_{{\updelta }}$ are eliminated by differencing the first order and our specification is optimized as follows:

2 $$ {\boldsymbol{n}}^{\cdot}_{it}=\alpha {\boldsymbol{d}}^{\cdot}_{it}+\gamma {\boldsymbol{in}}^{\cdot}{\boldsymbol{c}}_{it}+\lambda {\boldsymbol{n}}^{\cdot}_{it-1}+c_t+\varepsilon^{\cdot}_{it}\;$$

In the resulted specification, the variables of the model represent the first difference of the corresponding logarithm forms and $\upepsilon ^{\cdot}_{it}$ is outcome of first-order difference of error term $\upepsilon_{it}$. Besides, the ${\text{Z}}_{\text{t}\left[1,\text{k}\right]}^{{\prime }}$ is reduced to fixed time effects ${ c}_{t}$. Also, to tackle with the endogeneity and addressing the unobserved heterogeneity, we added a lagged dependent variable as an instrumental variable and we specified a dynamic panel data model. The same logic was applied on the other two models:

3 $$ {\boldsymbol{s}}^{\cdot}_{it}=\alpha {\boldsymbol{d}}^{\cdot}_{it}+\gamma {\boldsymbol{in}}^{\cdot}{\boldsymbol{c}}_{it}+\lambda {\boldsymbol{s}}^{\cdot}_{it-1}+c_t+\varepsilon^{\cdot}_{it}\;$$

4 $$ {\boldsymbol{f}}^{\cdot}_{it}=\alpha {\boldsymbol{d}}^{\cdot}_{it}+\gamma {\boldsymbol{in}}^{\cdot}{\boldsymbol{c}}_{it}+\lambda {\boldsymbol{f}}^{\cdot}_{it-1}+c_t+\varepsilon^{\cdot}_{it}\;$$

### Empirical specification and estimation

OLS-pool model is the most straightforward and most standard approach to estimate the coefficient of a panel data model. Nevertheless, this method produces sharply biased estimates if there is province heterogeneity [24]. Based on Arellano and Bover [25] and Blundell and Bond [26] recommendation, the generalized method of moments system (GMM-SYS) is the best alternative approach that is consistent, asymptotically efficient, and provide a strong instrument for dynamic panel data which has more than three time-series observations. Because, GMM-SYS model uses the lagged dependent variable as an instrument from more than two lag series. Also, lags of dependent variable will highly correlated with lagged dependent variable but uncorrelated with the composite disturbance, if ${{\upepsilon }}_{it}$ is a white noise.

To test the validity of GMM assumptions, we used the m1 and m2 tests to check the conditions of first and second-order of serial correlation of the estimated residuals, respectively, and applied Sargan test, which checks the validity of the instruments used. Also, the Hansen test is used to test for the overall effectiveness of all the instrumental variables, and the Wald Chi-Squared test is used to check a possible heteroskedasticity of residuals if we obtain a regression model with fixed effects. All statistical analyzes were performed using STATA version 13 (Stata Crop LP, College Station, TX, USA).

The Research Ethics Committee approved this study protocol at the health equity research center, Tehran University of Medical Sciences and National Institute for Medical Research Development (No. NIMAD.REC.1397.290) and was found to comply with ethical standards.

## Results

The monthly-province level statistics of physician’s steady-state activities in KRS from 2014 to 2018 are presented in Table 1, which includes variables in level and growth rate; the number of surgeries, cost of each operation, population-adjusted number of the surgery, and the number of the surgery per active surgeon. It should be noted that by standardizing the operating costs in terms of the growth rate approved by the government, the effect of increasing the annual tariff is eliminated.

**Table 1:** The monthly-province level population characteristics; 2014-2018 (*N*=15729)

Years	2014	2015	2016	2017	2018	Growth rate
Statistics	Mean (SE)					Mean
***N***	11.03 (2.59)	12.48 (2.66)	14.83 (2.71)	16.27 (3.05)	18.19 (3.19)	64.91%
P_*n*_	1759.54 (75.02)	1793.20 (68.39)	1810.49 (77.04)	1884.47 (61.36)	1940.19 (55.51)	10.27%
P_*p*_	0.19 (0.02)	0.23 (0.02)	0.26 (0.02)	0.32 (0.02)	0.37 (0.02)	94.74%
P_*ph*_	1.39 (0.05)	1.44 (0.05)	1.53 (0.05)	1.65 (0.06)	1.88 (0.06)	35.25%

*N* number of KRS at monthly-province level, P_*n*_ cost of each surgery, P_*p*_ number of the surgery per 100,000 population, P_*ph*_ number of the surgery per active physician

The mean (median) monthly number of KRS in each province in 2014 was 11.03 (3), which increased by 64.91 % (67.00) to 18.19 % (5) in 2018. However, the population and physician-adjusted mean growths in these five years were 94.74 (71.43 %) and 35.25 (44.63), respectively. All the variables are positively skewed, as their mean is significantly larger than the median value. The cost of each KRS, depending on the type of operation (total versus partial knee replacement), the surgical approach (traditional surgery/minimally invasive surgery [quadriceps-sparing or lateral approach]), and the type of implant used, can vary greatly, which is confirmed by statistics depicted by ${\text{P}}_{n}$ variable in Table 1.

Table 2 presents the results of a panel-data unit root estimation based on the Augmented Dickey-Fuller Fisher test. The outcomes indicate that the non-stationary null hypothesis is rejected at the 1 % significance level for all variables. So, it can be said that all variables are integrated of the first order, which justifies the use of the GMM-SYS estimator.

**Table 2:** Panel-data unit root test (Fisher type based on Augmented Dickey-Fuller)

Variables	Inverse chi-squared *P*	Inverse normal Z
***n***_***it −*** **1**_	665.66^**^	-22.50^**^
***s***_***it −*** **1**_	520.99^**^	-19.22^**^
***f***_***it −*** **1**_	395.89^**^	-15.85^**^
***n***_***it***_	645.13^**^	-22.20^**^
***inc***	124.21^**^	-9.47^**^

^**^Indicate that the non-stationary null hypothesis is rejected at the 1% significance level

The findings of OLS-pool and two-step GMM-SYS regression models are reported in Table 3. The first two columns show the results of the standard OLS-pool regression, and the second two columns present the results of two steps of GMM-SYS regression. The dependent variables in columns (1) and (4) are the average number, in columns (2) and (5) are the volume, and in columns (3) and (6) are the weighted values of the services. The results of OLS-pool regressions provide evidence that all variables, except for the factor in columns (1) and (3), have the expected sign and are highly statistically significant at the 1 % level.

**Table 3:** Estimates of OLS-Pool and Two-Step Difference GMM models

Independent Variables	OLS-Pool			Two-Step Difference GMM		
	$\dot{{\mathbf{y}}_{\mathbf{it}}}=\dot{{\mathbf{n}}_{\mathbf{it}}}$ (1)	$\dot{{\mathbf{y}}_{\mathbf{it}}}=\dot{{\mathbf{s}}_{\mathbf{it}}}$ (2)	$\dot{{\mathbf{y}}_{\mathbf{it}}}=\dot{{\mathbf{f}}_{\mathbf{it}}}$ (3)	$\dot{{\mathbf{y}}_{\mathbf{it}}}=\dot{{\mathbf{n}}_{\mathbf{it}}}$ (4)	$\dot{{\mathbf{y}}_{\mathbf{it}}}=\dot{{\mathbf{s}}_{\mathbf{it}}}$ (5)	$\dot{{\mathbf{y}}_{\mathbf{it}}}=\dot{{\mathbf{f}}_{\mathbf{it}}}$ (6)
$\dot{{\mathrm{d}}_{\mathrm{it}}}$)SE(	0.71^a^ (0.03)	0.79^a^ (0.03)	0.07 (0.01)	0.94^a^ (0.05)	1.01^aa^ (0.04)	0.06^a^ (0.02)
$\dot{{\mathrm{y}}_{\mathrm{it}-1}}$ )SE(	0.63^a^ (0.02)	0.63^a^ (0.02)	0.68^a^ (0.03)	-0.04^a^ (0.05)	0.07^a^ (0.03)	0.11^a^ (0.05)
$\dot{\mathrm{inc}\ }$)SE(	0.15 (0.08)	0.09^a^ (0.07)	-0.04 (0.02)	-1.25 (1.88)	0.80 (0.33)	0.54 (0.35)
Constant	7.96^a^ (1.51)	13.06^a^ (1.69)	4.35^a^ (0.85)	36.18^a^ (32.56)	11.85^a^ (10.96)	0.84^a^ (5.94)
N. observation	823	823	823	823	823	823
R-squared	0.826	0.805	0.473			
F-test (*p*-value)				0.000	0.000	0.036
Wald test				601.34	599.96	8.55
Hansen test				0.361	0.284	0.413
Sargan test				0.277	0.175	0.339
M2 test				0.671	0.589	0.712

^a^Indicate that the coefficients are significant at the 1% level

The alternative regression models, the Two-step GMM-SYS approach, which is applied to avoid endogeneity biases and omitted variables and provides a short-run demand elasticity, confirm the previous outcomes except for factors in all models. The findings reveal that the physician density and lagged dependent factors significantly affect demand for KRS in Iran. Unexpectedly, the results suggest that the patients’ income level is not an essential feature in demand for the surgery.

The F-test results indicate that the GMM-SYS model is significant at the 1 % level and the Wald test does not show the presence of heteroscedasticity, and its Chi-square test statistic is significant at the 1 % or lower level in various model specifications. Also, the Hansen test does not reveal any problem about over-identification restrictions and confirms the validity of variables as instruments in the two-step GMM-SYS model. The Sargan test does not detect any correlation between the used instruments and the residuals. Finally, The M2 test confirms the absence of a second-order serial correlation of the residuals in the GMM-based regression.

## Discussion

Knee replacement surgery is a standard and very effective treatment option for managing severe end-stage knee pain resulting from osteoarthritis, post-traumatic arthritis, and inflammatory arthritis [1]. However, a significant proportion of postoperative patients report persistent knee pain, poor knee function, and patient dissatisfaction [1, 27, 28]. Over the past four decades, there have been numerous innovations in total knee replacement design and implantation techniques, prosthesis diversity, and even alternative treatment scenarios with lower cost or lower revision risk [29]. In situations where the surgeons act as a patient’s perfect agent, the choice between these options is based on provider discretion and patient preference. Otherwise, service providers choose the type of treatment scenario based on non-therapeutic factors, such as financial incentives [30]. In this study, we tested the possibility of PID for KRS in the Iranian health system by two separate econometrics models.

Both analytical models obtained the elasticity of number of KRS variable ($\dot{{\text{n}}_{\text{i}\text{t}}}$) as positive and less than one. This value was 0.71 and 0.94 in the OLS, and GMM-SYS approaches, respectively. With the increase in the surgeons’ density, the number of performed surgeries has also increased significantly at a 1 % level. This finding indicates demand rationing so that the average number of performed KRS by each surgeon has increased over time.

The service size variable (${\boldsymbol {s}} ^{\cdot}_{it}$) elasticity in both static and dynamic equations was obtained as 0.79 and 1.01, respectively. These values were higher than the elasticity obtained for the ${\boldsymbol {n}} ^{\cdot}_{it}$ variable. These positive differences are confirmed by specification models (3) and (6) for the services relative value factor in (Table 3). Generally, the outcomes show that with increasing orthopedic surgeons to population ratio, both the number and size of KRS services were increased significantly at 1 % level. The positive elasticity associated with the service number variable may be due to the availability effect, but the increase in the service size certainly cannot be related to this issue.

Therefore, the researchers conclude that even if we attribute the increased number of surgeries performed per surgeon to the availability effect, we still have a PID for the KRS in Iran, at least as much as the elasticity obtained for the relative value of the service (${\boldsymbol {f}} ^{\cdot}_{it}$). If we accept the GMM-SYS model as an appropriate approach, the PID is about 6 %.

The observed positive association between physician to population ratio and the number of the KRS service per physician is contrary to that of Delattre and Dormont [22] which studied the behavior of general and specialist physicians in France and that of Redisch et al. [31] that analysis the PID among Unites States general physicians. However, they presented the same final results; the existence of a significant PID. Carlsen and Grytten [32], Sorensen and Grytten [23], Grytten and Sorensen [33] in similar studies did not show an established PID among Norwegian primary care physicians. These differences may be attributed to the difference in the health system structure, the type of payment system, the existence of a fixed or flexible reimbursement fee, and the type of services studied. In those health systems with managed care, apply flexible fees and use the Fee-For-Service method to reimburse the providers, creating a PID is much higher.

Iranian health insurances cover up to 90 % of the inpatient services costs, such as KRS in the public sector, but are highly inefficient in the private sector. Therefore, the financial burden of this PID will be on both the buyers and the recipients of the service. It should be noted that the value of 6 % is the minimum PID for KRS and may be much higher, which this study is not able to show its actual value. It seems that the main reasons for the existence of PID for KRS service in Iran are the lack of a managed care system, not using the clinical guidelines, lack of adequate supervision of surgeons, the existence of Fee-For-Service payment system, and severe information asymmetry between the health insurances and the service providers. Therefore, to reduce the sizeable economic burden of PID on the health system, each of the above reasons should be considered and modified by the health policymakers. Besides, we emphasize the need for new strategies to treat early-stage osteoarthritis, ultimately reducing the demand for surgery.

The study data indicates unequal access to orthopedic surgeons across the provinces. On average, there are 1.16 (SD: 1.08) surgeons with a median of 0.98 (IQR: 0.80) per 100,000 population. This is while the lowest and highest values of this statistic belong to Lorestan (6.11) and North Khorasan (0.33) provinces, respectively, which have a difference of 18.52 times. Reducing this unequal distribution to provide better access for less privileged provinces can be very effective and should be considered by health policymakers.

## Conclusions

This is the first time that the existence of PID in the Iranian health system is investigated using approved econometric models. Although the increase in the ratio of orthopedic surgeons to the population increased patients’ access to KRS services, the econometrics results show the mean of service size had increased to a higher degree over time. This finding strongly supports the existence of PID for aggressive, costly, and high-risk services such as KRS. In other words, a significant part of the increased demand for KSR services was for PID.

Due to the high coverage of KRD service costs by the Armed Forces Insurance in both the private and public sectors, the financial burden of this induced demand is imposed on the insurer. Therefore, health policymakers can minimize this induced demand by setting stricter criteria for KRS licensing and requiring physicians to adhere to the relevant clinical guideline.

## Data Availability

The datasets generated and/or analyzed during the current study are not publicly available for confidentiality reasons since individual privacy could be compromised but are available from the corresponding author on reasonable request.

## Abbreviations

PID: Physician-Induced Demand
GMM-SYS: Generalized method of moment’s system
KRS: The Knee Replacement Surgery
DPD: Dynamic panel data
SE: Standard Error
IQR: Interquartile Range
